# Modelling the sensory space of varietal wines: Mining of large, unstructured text data and visualisation of style patterns

**DOI:** 10.1038/s41598-018-23347-w

**Published:** 2018-03-21

**Authors:** Carlo C. Valente, Florian F. Bauer, Fritz Venter, Bruce Watson, Hélène H. Nieuwoudt

**Affiliations:** 1 Distell, Stellenbosch, South Africa, Stellenbosch University, Stellenbosch, South Africa; 2 Institute for Wine Biotechnology, Department of Viticulture and Oenology, Stellenbosch University, Stellenbosch, South Africa; 3 Department of Information Science, Stellenbosch University, Stellenbosch, South Africa

## Abstract

The increasingly large volumes of publicly available sensory descriptions of wine raises the question whether this source of data can be mined to extract meaningful domain-specific information about the sensory properties of wine. We introduce a novel application of formal concept lattices, in combination with traditional statistical tests, to visualise the sensory attributes of a big data set of some 7,000 Chenin blanc and Sauvignon blanc wines. *Complexity* was identified as an important driver of style in hereto uncharacterised Chenin blanc, and the sensory cues for specific styles were identified. This is the first study to apply these methods for the purpose of identifying styles within varietal wines. More generally, our interactive data visualisation and mining driven approach opens up new investigations towards better understanding of the complex field of sensory science.

## Introduction

Recent years have seen the emergence of rapidly growing volumes of publicly available information on the sensory properties of foodstuffs^1–4^. The details are typically in text format in printed or electronic documents, books, and online social media such as food review blogs and repositories^5,6^. Parallel to this development is the growing trend to process and organise these often large and complex volumes of information into databases or data warehouses so that techniques can be applied to extract useful knowledge from the original sources^6,7^. A feature of databases compiled in this way is that they are described as being *unstructured*^6^. This term implies that the format in which the original details are captured is unstructured, as would be the case if no fixed format template to enter the data is used in the creation of the database. Another interpretation of *unstructured* implies that the generation of elements such as content, author, date and time of creation of the information is more or less happening at random^6,8^.

*Data mining* refers to the process of exploring data to generate new knowledge and insights that are useful for a particular domain^6^. These insights include the generation of hypotheses based on the data, which may then be tested using traditional analysis techniques. This is in contrast to the traditional approach of generating hypotheses incidentally, or from deep researcher-insight into the processes underlying the data. The rapid growth in the size and variety of datasets (known as *big data*) in the past decade has resulted in the emergence of new mining techniques and algorithms. Much of this big data (which forms the input of such algorithms) is *text* – and this subfield of data mining is often referred to *text mining*^6,9^, itself a relatively mature field. Such mining techniques broadly consist of a few phases, which may be used iteratively:

1. Text extraction, pre-processing and standardisation, in which the keywords and phrases in the unstructured text are extracted, along with their relationships within that text^6,9,10^. This phase often involves cleaning the data, for similar terminology, spelling mistakes, etc.
2. Correlation and classification^9,11,12^, in which the extracted concepts and their relationships are structured, perhaps hierarchically or as a graph, showing implications and other relationships. In some cases, this phase highlights remnants of the text, such as synonyms.
3. Visualisation of the correlations^9,11,13^, usually giving clear visual hints as to relationships and potential hypotheses on the data.

The first and third phases are well-explored^6,9,10,13^. There are numerous approaches for the second phase, including: decision trees, association rules, formal concept lattices, hierarchical cluster analysis, neural networks, classification techniques and machine learning, as recently reviewed^9–11^. Earlier work proposing the application of concept lattices to *knowledge discovery in databases* (KDD) established the basis for interactive data visualisation/mining used in further sections of this paper^12,13^. Data mining outcomes are used towards the improvement of production processes and products alike^14,15^. The value of this strategy is, therefore, particularly evident when the data mining is done to generate *domain-specific knowledge* – expertise derived from a broad-based understanding and experience of a particular industry, such as gastronomy and wine-informatics^2,3,5,16,17^.

One of the most comprehensive and data-rich sources of publicly available information on South African (SA) wines is the annually published *John Platter Wine Guide to South African Wines*, referred to as Platter’s in the text^18^. Platter’s was conceived in 1978 and contains yearly entries from some 1,300 SA wine cellars and 15,000 individual wines. Several hundred new wines are entered into the guide each year. A panel of Platter’s appointed experts with extensive domain knowledge is tasked to assess the wines for their sensory attributes and quality. Since the target readership of Platter’s is the general public, an unstructured approach of generating the sensory data is used. Hence, panellists are not given specific instructions or a pre-determined list of attributes from which to select appropriate descriptors to describe the wines under evaluation. Instead, they rely on their wine domain expertise and describe their own sensorial impressions of the wines under evaluation using visual cues, smell, and taste.

In recent years, there has been renewed research interest in the sensory characteristics of Chenin blanc within the broader context of other white wine varieties due to the economic importance of Chenin for the SA Wine industry. Collectively, the wine varietals Chenin blanc, Sauvignon blanc, and Chardonnay hold the largest share (per volumes produced) in the SA white wine category^19^. The grape is cultivated in diverse geographical regions across SA wine growing regions^20^ due to its high yields and adaptability to the relatively hot and dry climatic conditions prevailing in the country, occupying more than 18% of all SA vineyards^19^. The wines have also gained prominence in recent decades as result of a shift from bulk production towards ultra-premium quality table wines, as attested by numerous international awards earned by the category^21^. As a result, the figures of locally consumed and export Chenin blanc volumes have shown steady increases since 2002^19^. Nonetheless, the extant scientific literature on the sensory aspects of SA Chenin blanc wine is limited to a few, and in some instances old references that do not cover modern-day wines^22–24^.

According to wine journalists, South Africa produces Chenin blanc wine across multiple styles^21^, and previous sensory research aimed at characterising the sensory space occupied by the varietal was hampered by the limited number of wines that could be tested with classical sensory profiling methods^25,26^. In this study, we took a novel approach and mined the Platter’s data for more than 2,500 Chenin blanc wines that were produced over a 7-year period, 2008–2014. We report on a novel application of a data visualisation technique, known as *formal concept lattices* in the subfield of information science and machine learning^11,27^ to model the wine style patterns. Although the lattice projection method has been extensively applied in computational linguistics, cybersecurity, and recommender systems^11^, the work reported here is, to our knowledge, the first attempt to use the technique for visualisation of the sensory characteristics of wine. It is also the first attempt to model the sensory space of a wine varietal by mining unstructured and publicly available wine sensory text data.

Since we were aware that patterns emerging through modelling of the unstructured data might be mining or visualisation artefacts, they were expressed as hypotheses on the data. We also used the data on some 4,300 Sauvignon blanc wines published in Platter’s for the same period (2008–2014) in the lattice construction, since the sensory characteristics of Sauvignon blanc are well documented and based on established sensory and statistical methods^28–30^. Patterns and underlying dimensions in the Sauvignon blanc lattice could, therefore be validated by established statistical methods. The hypotheses were then tested with conventional statistical techniques, all of which have received a high level of exposure and application in studies concerned with the sensory perceptions of foodstuffs, including wine. Multidimensional scaling^31^ was used to obtain a visual representation of the relationship between the sensory attributes of the varietal wines. The main subgroups within the Chenin blanc space were further investigated with correspondence analysis (CA)^32,33^. The Classification and Regression Trees (CART) method^34,35^ was used to identify the predictor sensory cues for the different styles. Due to the nature of the dataset and the complexity of the unstructured matrix, text standardisation^36,37^ was applied before the data were mined.

## Results and Discussion

### Visualisation of the sensory spaces of varietal wines using formal concept lattices

The sensory descriptors included in this study were used by the Platter’s panellists to describe all the Chenin blanc and Sauvignon blanc wines entered into the Platter Guide from 2008 to 2017. Chenin blanc is entered into Platter’s in three style classes; unwooded dry (no noticeable wood sensory character and residual sugar content not exceeding 5 g/L); wooded dry (noticeable wood sensory character and residual sugar content not exceeding 5 g/L); and wooded or unwooded semi-dry (RS content more than 5 g/L and not exceeding 12 g/L). Sauvignon blanc is entered into two style classes; respectively unwooded dry or wooded dry.

The total number of words used by the Platter’s panellists’ to describe the 2,746 Chenin blanc wines (all styles combined) included in this study amounted to 38,503 and 71,892 words for 4,352 Sauvignon blanc wines (all styles combined).

**Figure 1:**
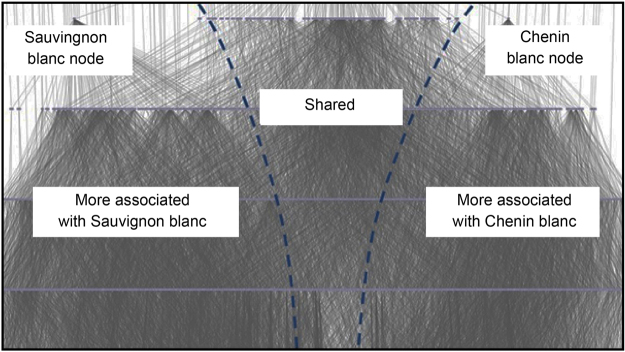
Top part of the full lattice constructed on the standardised sensory attributes of Chenin blanc (266 attributes) and Sauvignon blanc wines (250 attributes). The dashed vertical lines indicate rough boundaries between varietal discriminating clusters of concepts.

Lattices are constructed on c*ategorical data*, where the number of attributes are finite and can be labelled as *formal concepts*; as explained in the Methods sections. In this context, the standardised Platter’s descriptors were considered as formal concepts. These original words and terms were subjected to pre-processing and standardisation of terms, as described in detail in the methods and materials sections, using established procedures described in other studies^36,37^. Since we were only interested in aroma attributes, all non-sensory words were removed from the original data set, and duplicates were eliminated (for example *bubblegum* and *bubblegummy)*. Stop and function words were also eliminated. The final format consisted mostly of single words and in a few cases of bigrams where the pair of consecutive words described a single concept, for example *ripe fruit*, or *citrus fruit*. The outcomes of the cleaning-up stage of the text data were independently validated by three researchers working on the project. All the different aroma sensory words that were identified using this strategy, were used in the lattice construction shown in Fig. 1, and amounted to 266 and 250 different aroma sensory words for, respectively, Chenin blanc and Sauvignon blanc wines.

Both the sensory spaces of Chenin blanc wines and the Sauvignon blanc wines were easily identified in the lattice, since they were each hanging from a node, labelled respectively by the varietal. Although the publication lattice in Fig. 1 is too small to see specific attributes, interesting features were identified. Some sensory attributes located more towards the Sauvignon blanc side, and others more towards the Chenin blanc side of the lattice. The central region (labelled as *Shared* in Fig. 1) consisted of a large number of attributes that were common to both varietals. The edges (connecting lines between nodes) emanating below these common attributes were extensive, and these attributes covered most of the wines.

Attributes appearing further from the centreline (that is, more to the sides of the diagram) are more uniquely Sauvignon blanc or Chenin blanc. As such, those attributes provide increasing (depending on how far from the centreline) amounts of differentiation against the *other* varietal’s attributes. Attributes lower in the lattice (regardless of distance to the centreline) are more specific and apply to an increasingly smaller (depending on how low) group of wines. Conversely, attributes appearing high in the lattice are very general and apply to a larger group of wines, culminating in some of the top attribute nodes, such as ‘Sauvignon blanc’ and ‘Chenin blanc’. The lattice, therefore, visualises both the overall sensory space as well as the connections and hierarchy between all of the individual descriptors.

**Figure 2:**
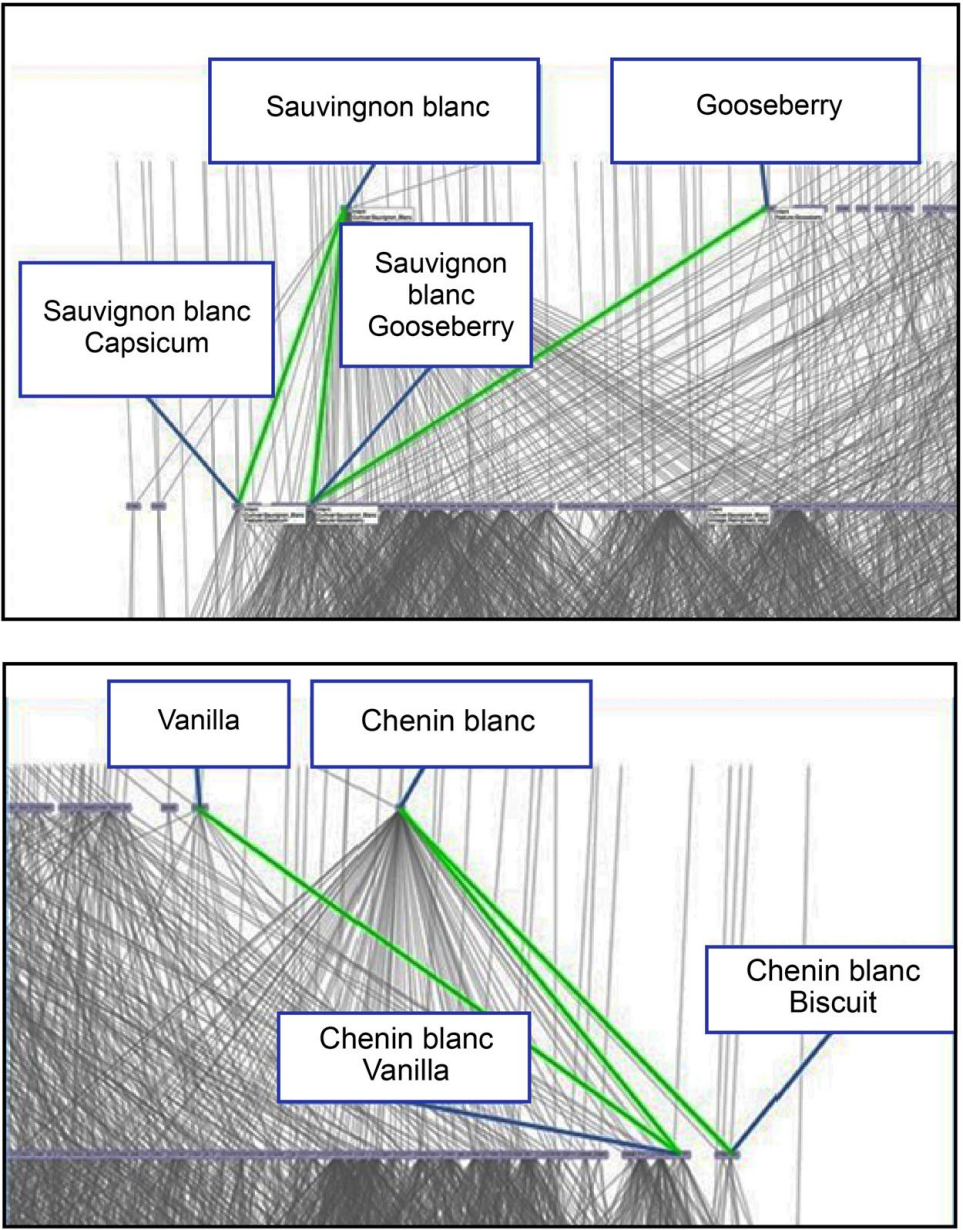
(**a** and **b**) Zoomed-in views of the Platter’s lattice. **(a)** Sauvignon blanc side, and (**b**) Chenin blanc side. Blue lines and text boxes indicate the identity and position of the nodes, while the green lines indicate the connection between nodes and sensory attributes.

The varietal discriminating sensory attributes were explored through zoomed-in views of the Sauvignon blanc and Chenin blanc sides of the Platter’s lattice. On the Sauvignon blanc side (Fig. 2a), the association between the attribute *capsicum* and this varietal was clear. *Gooseberry* was also associated with Sauvignon blanc but was also shared with Chenin blanc. The attributes *Capsicum* and *Gooseberry*, are typically associated with premium quality Sauvignon blanc wine^28,30^, and the lattice showed these attributes to locate towards the Sauvignon side of the projection. Also of interest is that *Capsicum* located to the extreme Sauvignon blanc side of the lattice, but on “lower level” than *Gooseberry*, therefore, it does not apply to as many wines as the *Gooseberry* attribute.

On the Chenin side of the Platter’s lattice (Fig. 2b) the attribute *Biscuit* located to the extreme side of the lattice, suggesting that this attribute is unique for Chenin blanc wines. *Vanilla* is a secondary aroma attribute resulting from the maturation of wine in contact with wood and it is to be expected that this attribute would be present in the wooded styles of both varietals. It was of interest that *Vanilla* also appeared higher up in the lattice, and on the same level as the Chenin blanc node, indicating that this is a very general attribute that occurred in both varietals, as would be expected for the wooded styles. The number of wooded wines in the Chenin blanc category was much higher than in the Sauvignon blanc category for example, in the dry wine categories of 2009, a total of 207 unwooded and 110 wooded Chenins were entered, in comparison to 544 unwooded and 24 wooded Sauvignon blanc wines. The positioning of the *Vanilla* attribute towards the Chenin side of the lattice was, therefore, to be expected.

**Figure 3:**
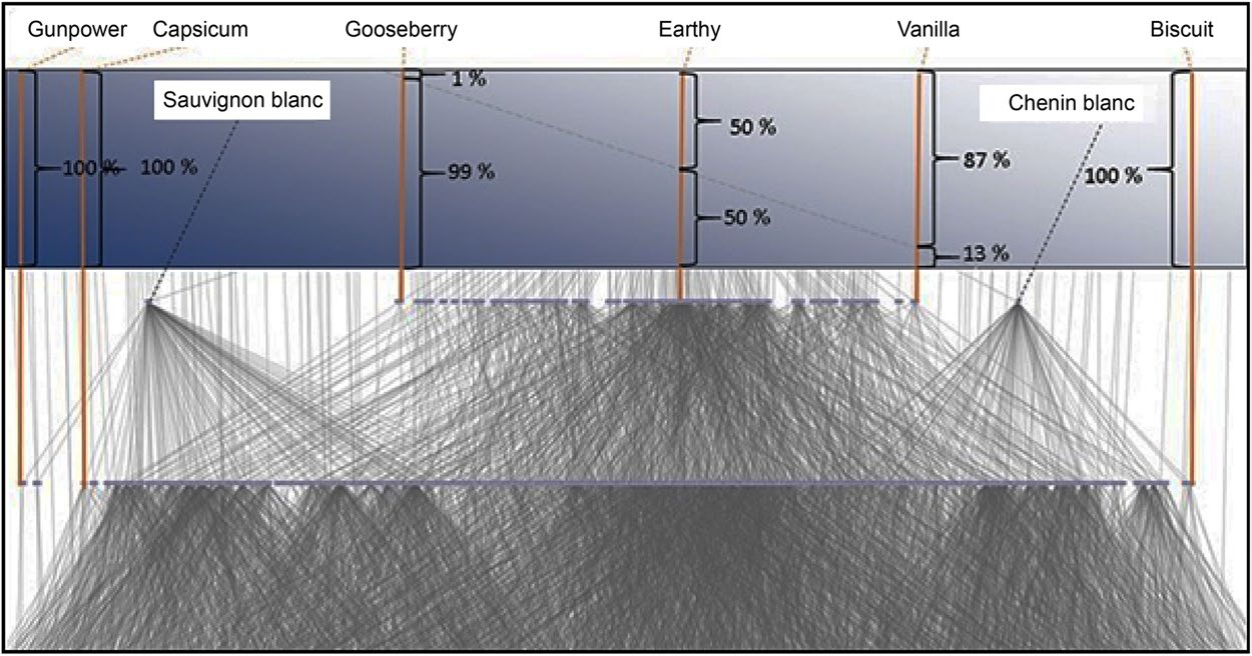
An illustration of the strength of association between a few selected aroma attributes and the wine varietals Sauvignon blanc and Chenin blanc.

An illustration of the strength of association between a few selected aroma attributes and the wine varietals is shown by the sliding scale in Fig. 3. The attributes *Gunpowder* and *Capsicum* were uniquely associated with Sauvignon blanc and *Biscuit* with Chenin blanc. *Earthy* was associated equally strong with both varietals, while *Gooseberry* and *Vanilla* were both shared attributes, but more strongly associated with respectively, Sauvignon blanc and Chenin blanc.

Although we interpreted the results as a supportive of the validity of the visualisation of the unstructured text data using the formal lattice concept strategy, it should be kept in mind that there is one important caveat regarding such data-driven science: generated hypotheses are the result of algorithmic (and human) *pattern-finding* in the data, and non-existent patterns may be found, meaning that all hypotheses should be tested with established statistical techniques, as we describe in the following sections.

### Validation of Platter data through classical statistical analysis

Since we were also interested to explore the Platter’s dataset for evidence of style patterns within the white varietals, we applied traditional statistical methods to the standardised text data. For this purpose, and for the sake of clarity, we only included terms that were mentioned 50 times or more in the standardised data. Table 1 shows the attributes that were cited more than 50 times over the 7 years investigation period.

**Table 1:** Standardised aroma sensory attributes for Sauvignon blanc (n = 39) and Chenin blanc (n = 39) that were mentioned 50 times or more over the investigation period of 2008–2014.

Sauvignon blanc		Chenin blanc	
Attribute (Number of citations)	Attribute (Number of citations)	Attribute (Number of citations)	Attribute (Number of citations)
Mineral (662)	Steely (110)	Oak (553)	Thatch (111)
Tropical (660)	Melon (108)	Acid/Acidity (525)	Mineral (107)
Grass (581)	Pineapple (106)	Apple (440)	Sweet (106)
Gooseberry (378)	Herbal (103)	Fresh (395)	Citrus (102)
Fig (349)	Greengage (95)	Tropical (307)	Complex (100)
Lime (292)	Leaves (90)	Dry (300)	Creamy (97)
Fruity (262)	Lemongrass (88)	Fruit (297)	Spice (97)
Passionfruit (240)	Herbs (88)	Ripe fruit (283)	Semi-dry (93)
Green (234)	Granadilla (83)	Rich (263)	Apricot (92)
Flint (205)	Pear (81)	Melon (242)	Lemon (86)
Green pepper (195)	Fynbos (81)	Crisp (232)	Quince (85)
Asparagus (192)	Blackcurrant (80)	Balance (214)	Juicy (84)
Grapefruit (188)	Savoury (77)	Peach (183)	Round (80)
Nettle (175)	Pithy (66)	Honey (172)	Vanilla (77)
Herbaceous (171)	Khaki Bush (60)	Light (168)	Full (70)
Apple (167)	Guava (54)	Pear (149)	Savoury (68)
Lemon (155)	Oak (53)	Lees characteristics (148)	Nuts (63)
Dust (143)	Green peas (52)	Pineapple (136)	Guava (62)
Capsicum (128)	Floral (52)	Floral (131)	Almond (52)
Citrus fruit (124)		Lime (113)	

**Figure 4:**
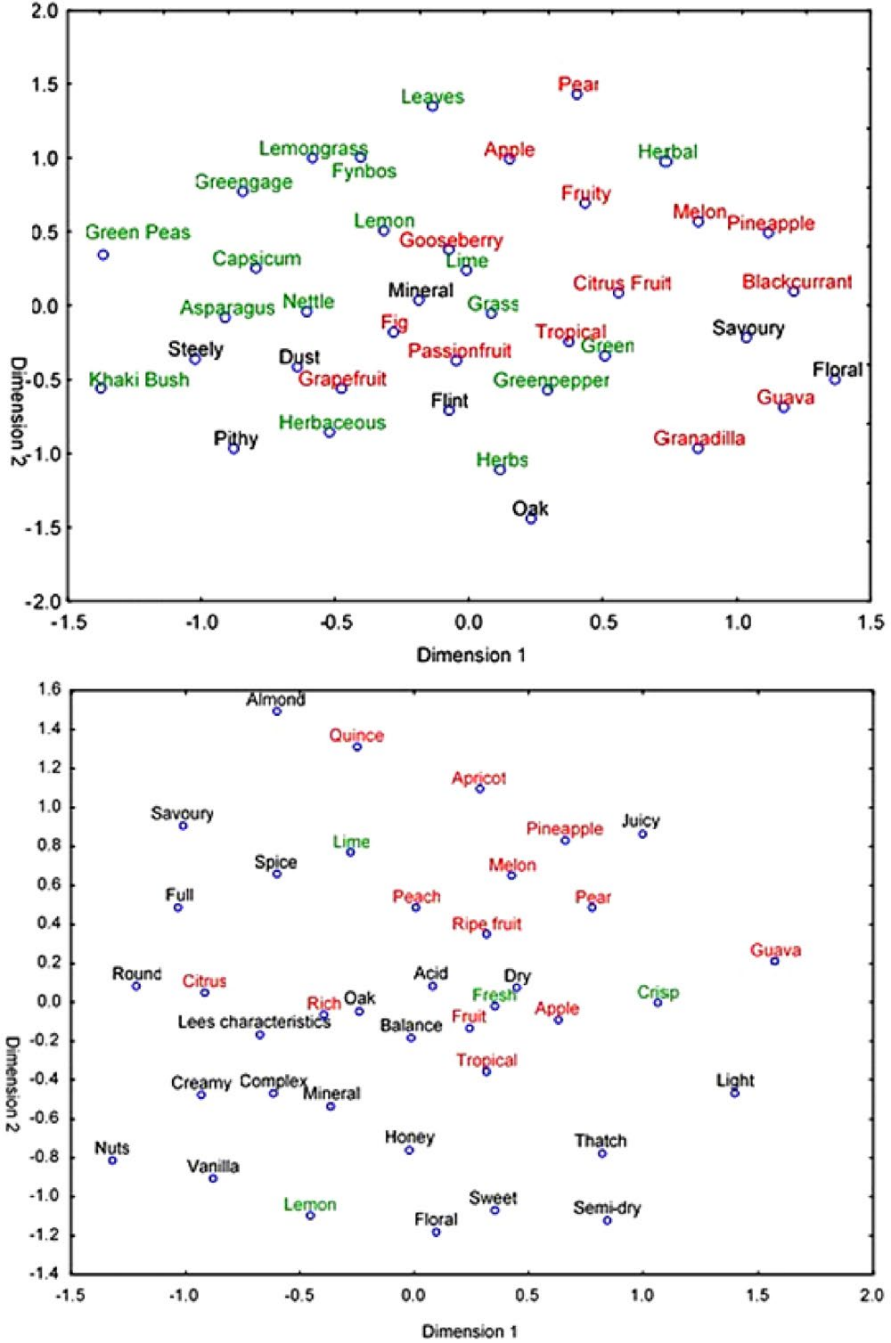
(**a** and **b**) MDS analysis of (**a**) Sauvignon blanc and (**b**) Chenin blanc wines based on sensory attributes mentioned more than 50 times. An explanation for words in green and red font is given in the text.

These attributes were subjected to MDS to obtain a multi-dimensional projection of the association between the sensory attributes within each varietal. We used the guidelines for standardised wine aroma terminology^38^ to interpret the MDS plots that were constructed on the data presented in Table 1. On the left-hand side of the plot (Fig. 4a), the herbaceous/vegetative spectrum (words coloured in green) was evident with attributes such as *Green peas*, *Asparagus* and *Khaki bush*. The right-hand side of the plot showed the fruity end of the spectrum with *Pineapple*, *Guava*, *Melon*, and *Blackcurrant* being typical of the tropical Sauvignon blanc wines (words coloured in red). These two poles, respectively an herbaceous/vegetative category and a fruity category were interpreted as distinctly different wine styles. As previously stated, Sauvignon blanc is well documented as having two main sensory styles^30,39^. We interpreted our results as a clear indication that the large body of unstructured sensory text data in the Platter’s guide could be processed and mined to extract meaningful wine domain-specific information for Chenin blanc, as a first attempt to define the sensory boundaries of this varietal.

For the Chenin blanc wines (Fig. 4b) it was difficult to identify main sensory directions from the MDS analysis, since distinct *poles* or attribute categories, were not obvious. SA Chenin blanc wine has been described as having a diversity of styles^21^ and our results could be a confirmation of this popular notion. On the other hand, it should be kept in mind that we limited the sensory attributes in this first data mining attempt to aroma attributes only, and the inclusion of taste attributes to enrich the data, should be done in future work.

**Figure 5:**
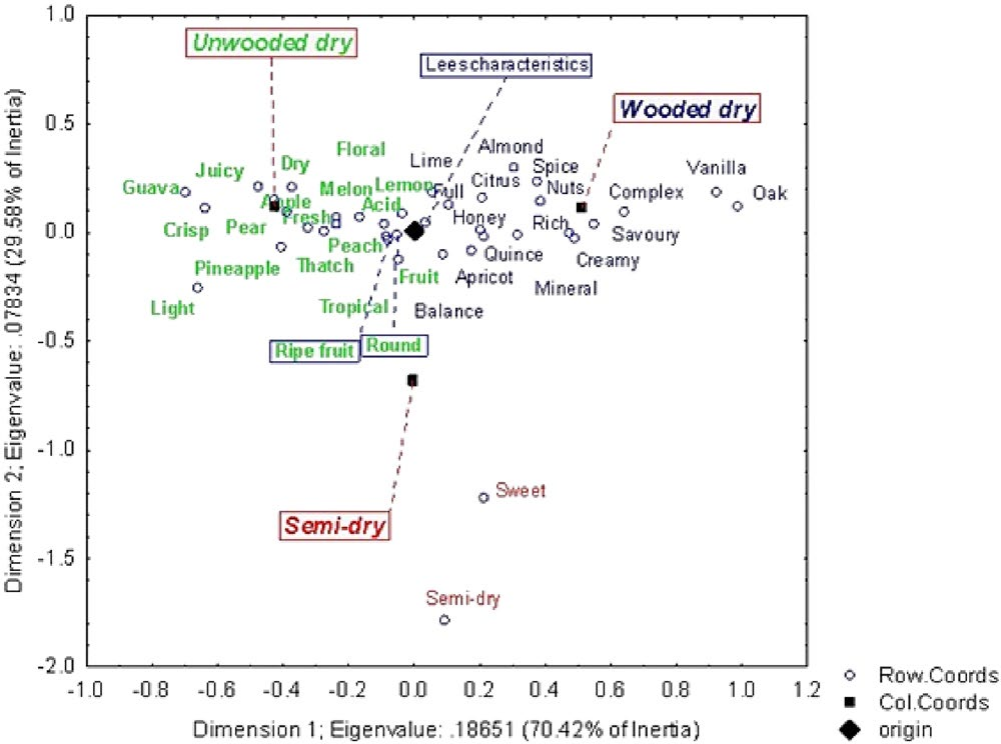
Correspondence analysis of the sensory attributes for Chenin blanc against style classification as proposed by the Platter’s data set. Some labels were slightly moved for the sake of clarity. These are connected with a dotted line to the original position on the graph, indicated by a small circle.

In order to investigate how the sensory attributes corresponded with the style categories used for Chenin blanc wines in Platter’s (respectively unwooded dry, wooded dry and semi-dry) CA was done and the results shown in Fig. 5.

The attributes *Sweet* and *Semi-dry* clearly corresponded to the semi-dry wine category as would be expected. The left-hand side of the plot was dominated by *Crisp*, *Light* and some fruity attributes including *Guava, Pear*, *Pineapple*, while the right-hand side of the plot was dominated by attributes associated with wood maturation, *Oak*, *Vanilla*, and *Complex*. This observation would suggest that in its simplest form, the main sensory dimension in SA Chenin blanc wine is related to *complexity*, with a seemingly spectrum ranging from fresh and crisp wine styles towards complex wines with noticeable wood character. This observation is consistent with our previous results obtained for Chenin blanc using descriptive analysis and trained panels, even though the previous work was done using much smaller sample sets^25,26^. From a wine domain-specific perspective, it was also of interest to note that the attribute *Lees Characteristics*, which refers to the sensory attribute developing in wine through extended contact with the wine yeast during the wine-making process, located mid-way between the two poles, *Crisp*, *Light* versus *Oak*, *Vanilla*, and *Complex*. We interpreted this observation as supportive of our interpretation that *complexity* is an important driver of Chenin styles.

### Sensorial cues for Chenin blanc and style classification CART test

**Figure 6:**
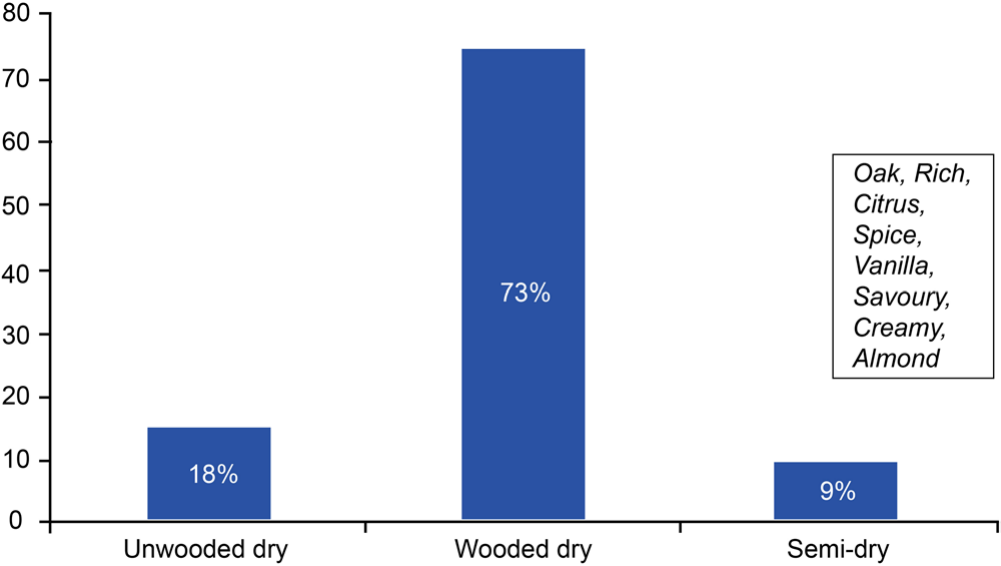
Bar graph showing how attributes *Oak*, *Rich*, *Citrus*, *Spice*, *Vanilla* and *Nuts* are related to the Chenin blanc wine styles.

It was of interest to determine whether mining of the Platter data could lead to the identification of the sensory cues for the Chenin blanc dry wine categories, and more specifically, if the style of a wine could be predicted based on the sensory attributes. For this purpose, a CART test was done to identify those attributes most likely associated with the wooded dry (Fig. 6) Chenins.

When the attributes *Oak*, *Rich*, *Citrus*, *Spice*, *Vanilla*, *Nuts*, *Creamy*, *Savoury*, and *Almond* were used to describe the wine it was associated with being an unwooded Chenin blanc in 18%, and an semi-dry Chenin blanc, 9% of the instances, while it would be a wooded Chenin blanc in 73% of the instances. These results gave us a fairly clear-cut sensory picture of the wooded wines’ cues.

**Figure 7:**
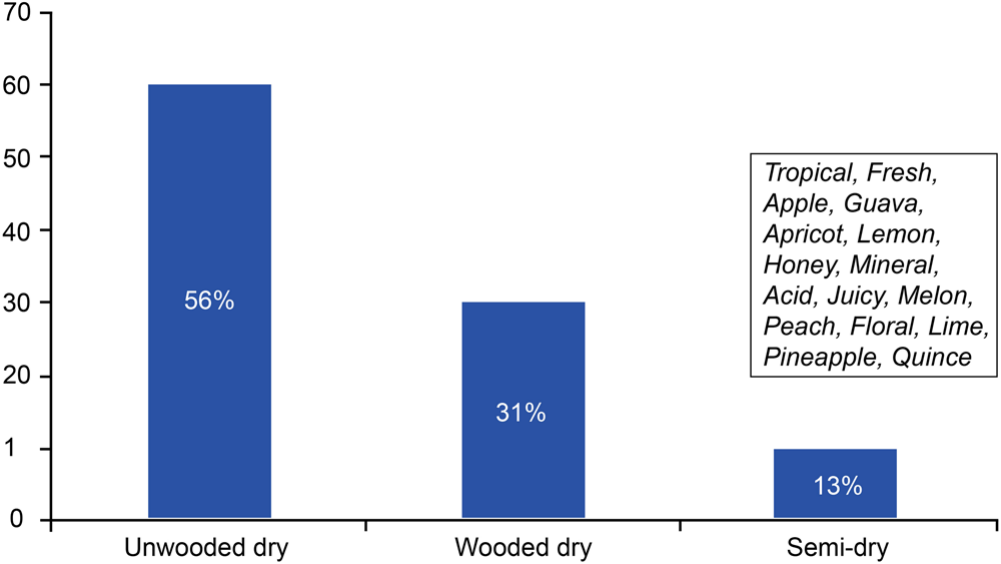
Bar graph showing how attributes *Tropical*, *Fresh*, *Apple*, *Guava*, *Apricot*, *Lemon*, *Honey* and *Mineral* are related to Chenin blanc wine styles.

When observing the CART results for the unwooded dry Chenin wines (Fig. 7) the grouping of attributes seemed to be more complex than that of the wooded style. More attributes, than for the wooded style, were required to characterise the unwooded style, and the classification was not as convincing as for the wooded styles. When observing the attributes *Tropical, Acid/Acidity, Balance, Fresh, Juicy, Crisp, Melon, Peach, Ripe fruit, Apple, Guava, Floral, Apricot, Dry, Pear, Thatch, Round, Fruit, Lime, Light, Honey*, *Lees characteristics, Full, Lemon, Pineapple, Quince* and *Mineral*, they were associated with unwooded Chenin blanc 56% of the time, 31% of the with wooded Chenin blanc and only 13% of the times with a semi-dry Chenin blanc wine. These results gave us the first real indication of the sensorial cues for the Chenin blanc unwooded style. The semi-dry style (graph not shown) could be characterised by two attributes, *Sweet* and *Semi-dry*, which were associated with the semi-dry style 61% of the time.

In summary, the mining of big unstructured text dataset of the Platter’s wine sensory descriptions resulted in the visualisation of the sensory space of SA Chenin blanc wine through our lattice data visualisation and mining methods, in combination with traditional statistical methods. Our novel application of lattice analysis to wine sensory data, successfully modelled the more than 500 sensory descriptors of the two white wine varietals, Sauvignon blanc and Chenin blanc. A large overlap of descriptors between the varietals were observed, while aroma sensory attributes that are unique to, or strongly associated with either group, were identified. From wine domain-specific perspectives, valuable information and insights were extracted, particularly in the identification of *complexity* as an important driver of Chenin blanc wine styles. Sensory cues for the Chenin styles were identified, and taken together, these findings have important implications for SA Chenin blanc wine style labelling. More generally, this work shows how the interactive data visualisation and mining methods can be applied to extract useful insights in other areas such as sensory science.

## Methods

### Platter’s Wine Guide

Platter’s contains information on thousands of wines of all varieties and styles that are annually produced in South Africa. Wine producers and *negociants* submit their wines of a particular vintage to a panel of industry experts, who are annually appointed to taste and evaluate the wines. Panellists generate a short description (10 to 40 words in English) of the perceived sensory attributes and give a quality rating out of five stars (with a score of five stars for the best quality) for each wine. Wine-specific metadata including producing cellar, region, grape variety, vintage, price, and bottle closure type is also captured and stored in a SQL database. The information is annually published in electronic format as Apps for iPhone/iPad and Android devices, and as hardcopy pocketbooks that are available from bookstores and other outlets.

### Protocols for sensory and quality evaluations of wines by Platter’s panellists

Cellars from all SA wine producing regions can enter either tank samples or bottled wines to Platter’s for assessment by an appointed panel, which varies in numbers from 15 to 20 people per year. The annual wine tastings take place from August to September and panellists taste the wines in the comfort of their homes, to ensure a relaxed environment and to avoid sensory fatigue. A single panellist tastes all the wines of a particular winery for a maximum period of three years. No pre-determined list of wine sensory attributes is provided to the panellists; rather, they can freely choose words and terminology to describe their perceptions of a particular wine in terms that are unrestricted to sensorial attributes. Panellists are not calibrated to perceive the same aroma and describe it using the same terminology, in contrast to the protocols followed with classical sensory descriptive profiling methods^40^. Only one panellist normally evaluates a wine and descriptions are therefore based on individual opinions. Wines can be entered for evaluation once, except if a tank sample was entered the previous year and the wine was bottled before the next round of assessments; or if the wine has earned awards at other wine competitions after the Platter’s evaluation, indicating improved quality derived from extended aging. A star rating out of five is also given, and wines that receive a quality rating of four stars or higher are re-tested by a small panel to verify the rating^18^.

### Platter’s wine descriptions included in this study

The sensory attributes of all the varietal Chenin blanc and Sauvignon blanc wines annually entered into Platter’s over the seven-year period, 2008 to 2014, were included in this study. The scope of the study was limited to include only dry and semi-dry wines.

The term *varietal wine* refers to a wine that is labelled by the grape variety, such as Chenin blanc or Sauvignon blanc, and according to the labelling requirements for SA wine, at least 85% of that wine must be produced from the specified variety^41^. Other varieties used in the production of the wine, if any, do not need to be specified. A wine that was produced from a single grape varietal, may be labelled as such.

Wines are entered into Platter’s in categories based on the residual sugar (RS) content of the wines, as set out in the SA Liquor Products Act nr 60 of 1989^41^. The scope of the study was limited to two categories, namely dry (RS content not exceeding 5 g/L) and semi-dry wines (RS content between 5 and12 g/L).

These categories are further sub-divided in Platter’s into *unwooded* or *wooded*, as indicated in Table 2, with *unwooded* and *wooded* defined in sensory context, as respectively, not a noticeable, or a noticeable wood character. In total, 2,746 Chenin blanc and 4,352 Sauvignon blanc wines were tasted by the Platter’s wine panellists, with the breakdown per annum and style shown in Table 2. All the wines were included in this study. The numbers represented at least 85% of the total number of commercial Chenin blanc and Sauvignon blanc wines that were annually produced in South Africa in the period under investigation^19^ and were therefore considered representative of the two varietals.

**Table 2:** Numbers of varietal Chenin blanc and Sauvignon blanc wines, per category, which were evaluated by Platter**’**s South African Wine Guide panellists from 2008 to 2014, and included in this study.

Year	Chenin blanc				Sauvignon blanc		
	Unwooded dry^a,c^	Wooded, dry^a,c^ wooded	Semi-dry^b^ (uw & w)^c^	Total	Unwooded dry^a,c^	Wooded, dry^a,c^	Total
2008	210	94	32	336	514	20	534
2009	207	110	40	357	544	24	568
2010	212	117	42	371	572	32	604
2011	201	113	66	380	592	34	626
2012	209	122	77	408	614	45	659
2013	225	137	79	441	632	52	684
2014	224	157	72	453	612	65	677
Total	1488	850	408	2746	4080	272	4352

^a^dry: Residual sugar (RS) content not exceeding 5 g/L; ^b^Semi-dry: RS content more than 5 g/L and not exceeding 12 g/L; ^c^uw & w: unwooded and wooded.

### Pre-processing and standardisation of text data

The sensory descriptions and accompanying metadata of each wine were exported from SQL to Microsoft Excel 2013 to prepare the data for mining purposes. Words and phrases in the original text were not restricted to sensorial terms, and also included hedonic, emotional, occasional, technical and food pairing words and phrases. Since we were interested in extracting aroma sensory attributes, the original descriptions were first cleaned up manually by one researcher to remove non-sensory words, e.g., *ideal for picnics*, *summer wine*. The outcomes were verified independently by two other researchers working on the same project. Function or stop words, which included prepositions and articles, were also removed from the data, following the procedures described in other studies^36,37^. The descriptions were further processed to eliminate duplicates of words within the same description, as long as it did not cause loss of meaning; for example, if the same attribute was mentioned twice in the description of the same wine. In a few instances bigrams were kept when the pair of consecutive words described one concept, for example *ripe fruit* and *citrus fruit*. These concepts are frequently used in the domain of wine sensory description of white wine. Two researchers again independently validated the actions taken and outputs obtained from this clean-up stage.

In the next phase, the attributes were standardised by combining synonyms and limiting excessive repetitions to reduce the number of terms, as illustrated in Table 3. Additional challenges posed by the unstructured data were cases where the same attribute was implied, but various panellists used different words; for example, *passion fruit* and g*ranadilla*. In this study, comparative statements such as *more fruit compared to previous vintages* were not included as far as possible. One researcher manually processed the data and two more researchers working on the same project checked the outcomes independently. Final validation of the outcomes was reached through discussion between the three researchers until consensus was reached.

**Table 3:** Examples of standardisation of words and phrases used in Platter**’**s to describe Chenin blanc wines, for the purposes of data mining of the text^18^.

Variations of word or phrases in the original Platter’s wine descriptions^a^	Standardised word
green apple, ripe apple, golden delicious apple, granny smith apple, fleshy apple, crisp apple, apple fruit salad bruised apple, yellow apple, red apple	apple
bubblegum, bubblegum hint, bubblegummy	bubblegum
styling peach, peach concentration, peach nuances, peach flavours, peach kernel, peachy, flush, sweet-ripe, peachiness, peach toned, gentle peach, sun ripe peach	peach
uncomplicated grass, whafty grass, dusty grass, dry grass	grass
perfumed citrus, citrus notes, citrus flavours, citrus peel, citrus intensity, citrus concentration, citrus zest, fine citrus, lively citrus	citrus

### Clarification of words

Some of the words had either a negative or a positive connotation attached to them. For the purpose of this study, only positive connotations were used for analysis, as negative connotations would not serve the aim of modelling the sensory space of the varietals. The following words were standardised with this mind.

**Oak** – All descriptions that mentioned the presence of oak on the pallet or nose were included in the study.

**Acid** – The context in which the descriptor *acid* was used in the descriptions, was carefully considered. The term was only included in cases where the panellists commented on *good acidity* and their perception there of. Instances where the panellists mentioned that the wines were lacking acidity, were not used in this study.

**Fresh** – The term *fresh* was included in all cases where it was used as a descriptor on its own. If an attribute was described as being *fresh apple*, only the attribute *apple* would be used and the word *fresh* would be omitted.

**Rich** – The word *rich* was used as both an olfactory description and as a mouth-feel attribute. *Rich* was only included as an olfactory attribute if it represented the entire sensory blurb, for example *rich fruit*.

**Balance** – *Balance* was included as an overall sensory perception of the wine.

**Lees characteristics** –*Lees characteristics* was included if the sensory description indicated that the wine had lees treatment, or if the panellists indicated that lees treatment enhanced the wine, or if they perceived the effects of the treatment during sensory evaluation of the wine.

Another aspect that was considered related to the simplification of the data for the purposes of computation. This was challenging since by reducing complete sentences to single words or in a few cases bigrams, some original meaning may be lost and thus impoverishing the corpus, as illustrated by the examples in Table 4.

**Table 4:** Original sensory descriptions of two different Chenin blanc wines in Platter**’**s^18^.

Wine 1*: Easy going tropical quaffer perfect for summer lunches*
Wine 2*: Shy, but opens up to delicious ripe peach, apricot notes, oak aromas still prominent but will integrate. Complexity from four different vineyards, three soil types & differing ripeness levels, all artfully blended*

Descriptions for Wine 1 and 2 in Table 4 are extracts from the original raw data obtained from the Platter’s descriptions of two different Chenin blanc wines. During the data pre-processing for Wine 1, the only word selected for this study was *tropical*. For Wine 2, several words would be retrieved. When considering the words *peach* and *apricot*, it could be argued that these fruits are indeed tropical fruit, but because the word *tropica*l was not used, this would not add to its specific tally. Even though the two descriptions have a similar idea on the fruitiness of the wine, they are not tallied the same.

### Lattice construction

The approach used to explore relationships between wine attributes in this paper is partly based on a field of research called Formal Concept Analysis (FCA)^42^. The following sections introduce the basic constructs of this approach.

Across domains, many datasets consist of *objects* and *attributes*. A very simple example, the set of *humans* as the objects with *gender* and *hair colour* as the attributes is used for illustration in Table 5. Such datasets can be viewed simply as *object-attribute pairs*, such as *(Bruce, male)* and *(Bruce, grey)*.

**Table 5:** Basic dataset of *object-attribute pairs* using two objects and two attributes for illustration.

Object	Gender	Hair colour
Bruce	Male	Grey
Carlo	Male	Black

A more visual presentation of the original *object-attribute pairs* data would be an *object-attribute matrix*, as shown in Table 6.

**Table 6:** Basic dataset of *object-attribute pairs* rearranged in an *object-attribute matrix* (for illustration).

Object	Male	Female	Black hair	Grey hair
Bruce	X			X
Carlo	X		X	

The above representations give remarkably little direct insight into the latent structures in the data. Perhaps there is a correlation between gender and hair colour? Of course, a *conjectured correlation* can be statistically tested against the pairs, but this begs the question of how to generate correlation hypotheses. Information- and data-science put forth the new approach of generating the hypotheses from visualisations of the data – specifically visualisations which highlight the hidden structures in ‘unstructured datasets’.

One of the recent and most successful (but by no means the *only*) visualisation techniques involves a *lattice*, which is known as a *formal concept lattice* in the subfield of information science and machine learning^9,27^. Consider the following object-attribute matrix in Table 7 of animals with various biological attributes.

**Table 7:** *Object-attribute* matrix of animals with various biological attributes (for illustration).

	Needs water	Lives in water	Lives on land	Needs chloro-phyll	1 leaf germi-nation	2 Leaf germi-nation	Is motile	Has limbs	Suckles young
Leach	x	x					x		
Bream	x	x					x	x	
Frog	x	x	x				x	x	
Dog	x		x				x	x	x
Spike-weed	x	x		x	x				
Reed	x	x	x	x	x				
Bean	x		x	x		x			
Maize	x		x	x	x				

**Figure 8:**
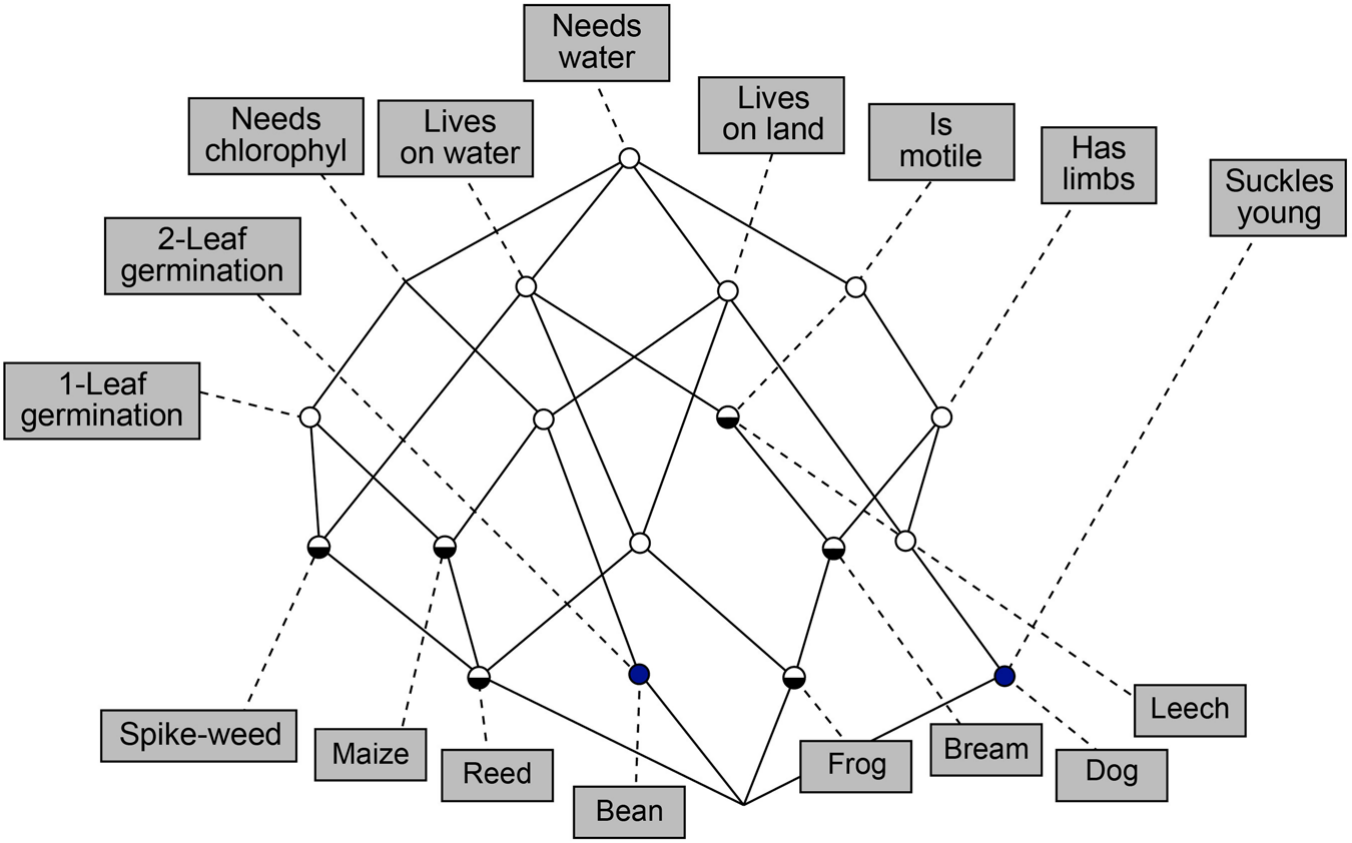
Formal concept lattice (or simply *lattice*) representation of some simple life-forms (nodes labelled by unshaded text boxes) and their attributes (nodes labelled by shaded text boxes). An attribute is *present* in all nodes hanging below it. Nodes without labels are *aggregating* attributes from their connections above.

This can be visualised as shown in Fig. 8.

The animals appear at some of the lower labelled nodes, while the attributes appear as labels on higher nodes. From the lattice, we can make several types of direct observations (which in larger lattices could be treated as hypotheses for testing), for example:

- The observation of something *with limbs* and *living in water* leads us unambiguously to *frogs*. This is surprising: from the matrix, one would have expected to need four attribute observations (including *Needs water* and *Is motile*) to reliably identify frogs.
- Reading downwards, the attributes of *Leaches* are entirely contained in those of *Bream*, which are entirely contained in those of *Frogs*.
- Similarly, reading upwards, 2-leaf germination implies both a need for chlorophyll and living on land.
- The common attributes of *Reeds* and *Frogs* are ‘Lives-in-Water’, ‘Lives-on-Land’ and ‘Needs-water’. We can see this by tracing from the reed and frog nodes upwards to their nearest common ancestor node, and then further upwards to all attribute nodes, giving these three attributes.
- *Leaches* and *Spike-weed* both live in water and need water.
- Attribute nodes appearing high in the lattice are more *general* than those appearing lower – they enjoy more use by appearing in more objects.
- The way the lattice is drawn (using a *graph drawing* algorithm) shows a certain amount of left-right symmetry – there is a ‘centreline’ to the lattice. Attributes further from the centreline are usually more specific; for example, ‘Has Limbs’ and ‘1-Leaf Germination’ are both highly specific, and far from the centreline.
- Visually, we can see a roughly 50/50 split between objects living in water versus those living on land, cf. the two attribute nodes high up on either side of the centre line.

In short, lattices are a form of *knowledge representation* of the dataset. As mentioned above, it can be used to conjecture hypotheses about the objects and attributes. All of the observations could have been made directly in the matrix format, but with considerably more effort. With large amounts of sensory data on wine, this simply would not be feasible.

There is one important caveat regarding such data-driven science: generated hypotheses are the result of algorithmic (and human) *pattern-finding* in the data, and non-existent patterns (visualisation *artefacts*) may be found, meaning that all hypotheses should be tested with established statistical techniques, as we describe in later sections.

### Building a lattice

As mentioned, lattices are a form of knowledge representation of the dataset. They are typically constructed automatically using one of many known *algorithms*, and there is substantial work on the mathematics and computational properties of lattices.

As a *classification and conjecture engine*, lattices are already powerful, however, applying a lattice to entirely new (not previously seen) data can yield interesting results and is a subfield of *machine learning* – itself a part of *artificial intelligence*. When faced with new data not falling directly into the lattice, the lattice may be *incrementally modified* with minimal effort – again using a well-known algorithm.

Lattices are a relatively large representation of the dataset (potentially requiring large computational resources to build) – an expected trade-off against the visualisation and pattern finding properties of lattices. In an application such as sensory attributes for wine, the lattice is usually built only once from an initial dataset, with small subsequent modifications, thereby amortising the construction computational cost.

### Statistical methods

The citation frequency for each cleaned-up and standardised sensory term was scored and entered in a two-way contingency table with the wines in rows and sensory attributes in columns. To analyse the text data with multi-dimensional scaling (MDS)^31,43^ a matrix was constructed based on the number of times that each pair of sensory words was mentioned together. The results were expressed as a percentage of the total number of times that at least one of the words in the pair was mentioned. The distance between two attributes can, therefore, be interpreted as a measure of them being mentioned together in the sensory descriptions. The style categories (dry, semi-dry, unwooded or wooded), were also entered for each wine. Correspondence analysis (CA), a multivariate method that investigates the symmetric correspondence or association between categorical variables^44^, was applied to the data to determine the association between the various wine style categories and sensory attributes. The non-parametric classification and regression trees (CART) algorithm^45,46^ was used to identify the predictor sensory cues for the dry, semi-dry unwooded and semi-dry wooded Chenin styles. An advantage of CART is that no assumption regarding the underlying distribution of the predictor variables is made and it can handle categorical predictors, such as the sensory attributes used in this study. All statistical procedures were done in Statistica version 13 (StatSoft Inc., Tulsa, USA).

### Validation of text pre-processing and lattice interpretations

The nature of the data used for this research precluded classical analysis of variance testing. However, when mining the unstructured sensory dataset where no instructions or guidelines were given to panellists to generate the data, questions arose whether valid and meaningful new knowledge could be extracted. Rigorous attempts were therefore made to validate the outcomes of the cleaning-up and standardisation of the original sensory descriptions. In all steps, one researcher manually did the data pre-processing, and the outcomes were checked independently by two additional researchers working on the same project.

The validity of our interpretations of the sensory space (and sub-spaces) of Chenin blanc wines visualised by the lattice was tested by including a second Platter’s dataset containing the sensory descriptions of 4,352 SA Sauvignon blanc wines that were evaluated in the same period (2008–2014) and by the same Platter’s panellists as the Chenin wines. We based our rationale for this approach on the well-researched and widely published sensory attributes of Sauvignon blanc wines produced in France, New Zealand, Australia and South Africa^28–30,39^. In addition, the final list of 266 Chenin blanc attributes that were extracted by the research this study were also presented to an independent panel of five Chenin blanc domain experts who checked that the final list of attributes extracted by the research, was relevant for this product category.

### Data availability statement

The authors declare that all materials and data are available.
